# Preoperative computed tomography-determined sarcopenia is a reliable prognostic factor in patients with gastric cancer after radical gastrectomy: A sex-specific analysis

**DOI:** 10.3389/fnut.2022.884586

**Published:** 2022-10-24

**Authors:** Ting Liu, Xiaoping Yi, Jie Ge, Jinwei Zhang, Fengbo Tan, Kun Song, Heli Liu, Mimi Tang

**Affiliations:** ^1^Department of Pharmacy, Xiangya Hospital, Central South University, Changsha, China; ^2^Institute for Rational and Safe Medication Practices, National Clinical Research Center for Geriatric Disorders, Xiangya Hospital, Central South University, Changsha, China; ^3^Department of Radiology, Xiangya Hospital, Central South University, Changsha, China; ^4^Department of Gastrointestinal Surgery, Xiangya Hospital, Central South University, Changsha, China

**Keywords:** gastric cancer, sarcopenia, survival, sex difference, body composition

## Abstract

**Background:**

The predictive role of sarcopenia in cancer prognosis is an area of increasing concern. However, the influence of sex difference on the predictive role of sarcopenia in cancer prognosis has not been clearly defined. This retrospective cohort study investigated the effect of preoperative sarcopenia on the long-term outcomes of patients with gastric cancer (GC) based on sexual dimorphism.

**Methods:**

Preoperative abdominal computed tomography (CT) scans from 379 GC patients who underwent radical gastrectomy were carefully analyzed. The patients were categorized into sarcopenia and non-sarcopenia groups according to the L3 skeletal muscle index (L3 SMI) measured on CT scans. Moreover, other indexes which can be used to evaluate the muscle area or the muscle quality, including skeletal muscle area (SMA), visceral fat area (VFA), subcutaneous fat area (SFA), skeletal muscle radiation attenuation (SM-RA), visceral fat index (VFI), subcutaneous fat index (SFI), and subcutaneous and visceral ratio (SV), were obtained from CT scans.

**Results:**

There were 254 men and 125 women included in our study. After calculation, we defined sex-specific SMI-related mortality cutoff as 39.73 and 32.97 cm^2^/m^2^ for men and women. Univariable analysis showed that pathological tumor-node-metastasis (pTNM), depth of invasion, lymph node metastasis, differentiation degree, preoperative sarcopenia (for men), SMA (for men), L3 SMI, SFA (for women), SFI (for women), SV (for women), and SM-RA (especially for men) were significant independent predictors of overall survival (OS). Multivariable analysis showed that pTNM, depth of invasion, poor differentiation, and SM-RA were significantly associated with 5-year OS in GC patients. However, CT-determined sarcopenia was associated with significantly worse OS only in men, and SFA was significantly associated with 5-year OS only in women.

**Conclusion:**

SM-RA is a reliable prognostic factor in patients with GC after radical gastrectomy. The impact of indexes mentioned above on survival outcomes is dependent on sex. CT-determined preoperative sarcopenia, a muscle-related indicator, was associated with outcomes in men. Adipose-related indicator (SFA), instead, was associated with outcomes in women.

## Introduction

Sarcopenia is defined as decreased muscle mass, quality, and strength in elderly people (1) and is closely associated with frailty syndrome in the elderly (2). As early as 2010, the European Working Group on Sarcopenia in Older People (EWGSOP) first advocated the diagnostic criteria of age-related sarcopenia (3), after which sarcopenia has aroused great concern, especially in cancer patients. Current research on the link between cancer and sarcopenia has focused on (1) the prevalence of sarcopenia in different cancers (4); (2) the predictive role of preoperative sarcopenia or postoperative sarcopenia in cancer prognosis (5); (3) radiotherapy-induced sarcopenia (6, 7); (4) the effect of sarcopenia treatment, such as exercise, nutrition, or medication intervention, on the prognosis or complications of cancers (8, 9); (5) the effect of sarcopenia on chemotherapy-related toxicity (10, 11); and (6) the potential mechanism of cancer-associated sarcopenia (12).

Gastric cancer (GC) is the third most commonly diagnosed cancer and the third leading cause of cancer death in China according to the latest epidemiological data (13). Surgical resection has been, without doubt, the most effective therapy for potentially curable GC we have had so far (14). As a previous report, the prevalence of sarcopenia in GC was 32.05%, which is the lowest one in digestive cancers (15–20), but higher than age-related sarcopenia (8.2% for men and 6.8% for women) based on EWGSOP algorithm (21). However, to date, there are no internationally accepted diagnostic criteria for sarcopenia in cancer patients. All the diagnoses concerning cancer-related sarcopenia are based on age-related sarcopenia established by EWGSOP or the Asian Working Group for Sarcopenia (22) or calculated by clinical data using statistical analysis (23, 24). Thus, current studies about the predictive value of sarcopenia in GC are still controversial.

Nowadays, several methods have been proposed for the assessment of muscle mass, including computed tomography (CT) scans, dual-energy X-ray absorptiometry (DXA), and bioelectrical impedance analysis (BIA). Among these, measurement of the cross-sectional muscle area at the level of the third lumbar vertebra (L3) on CT scans is the method that is used most commonly, since CT is a part of the routine staging process in cancer patients (25). In addition, muscle mass index (MMI) (16) which divides the patient’s muscle mass (kg/m^2^) or muscle area (cm^2^/m^2^) by the height squared is also widely used in the prognostic analysis of cancer.

Interestingly, a recent study from Korea suggested that the impact of postoperative muscle mass loss and surgery-induced sarcopenia on survival outcomes is dependent on sex. To be specified, the association between muscle mass and long-term survival was meaningful in men but not in women (26). Moreover, the amount and rate of muscle mass loss during aging also differ between men and women (27, 28), which suggests that we should consider the influence of sex factor when we investigate the relationship between sarcopenia-related factors and prognosis of GC. The reasons why gender influences prognosis may be complex, including gene expression (29), skeletal muscle kinetics, and fiber-type composition (30).

In this study, we present a retrospective investigation of the influence of sarcopenia on the prognoses of GC patients who underwent radical gastrectomy. In addition to the classic indicator L3 skeletal muscle index (L3 SMI), other indexes like visceral fat area (VFA), subcutaneous fat area (SFA), visceral fat index (VFI), subcutaneous fat index (SFI), subcutaneous and visceral ratio (SV), and skeletal muscle radiation attenuation (SM-RA) were also included in our study according to previous studies (31–35). Moreover, we further evaluated potential sex differences in the relationship between sarcopenia and long-term survival.

## Materials and methods

### Patients and procedure

The present study was approved by the ethics committee of Xiangya Hospital of Central South University. All included patients provided informed consent. Using a computerized database, we analyzed data on patients diagnosed with GC at the department of Gastrointestinal Surgery, Xiangya Hospital, Central South University, from January 2011 to December 2016. All patients with GC were confirmed by pathological examination.

The inclusion criteria were patients with (1) pathological stages I-III who accepted laparoscopic or open radical surgery; (2) no other chemotherapy or radiotherapy was implemented before the operation; (3) data integrity; and (4) no distant metastasis (M1). Patients with palliative care, gastric remnant cancer, and liver and kidney dysfunction were excluded.

### Data collection

Clinical data including age, gender, type of gastrectomy, pathological stage, degree of differentiation, and laboratory data [red blood cell (RBC), white blood cell (WBC), platelet (PLT), aspartate aminotransferase (AST), alanine aminotransferase (ALT), and creatinine (Cr)] were exacted from computerized database in the hospital. Survival status was obtained through follow-up by telephone. To assess for sarcopenia, we used adequate quality abdominal CT scan within 4 weeks before radical GC surgery.

### Skeletal muscle assessment

**FIGURE 1:**
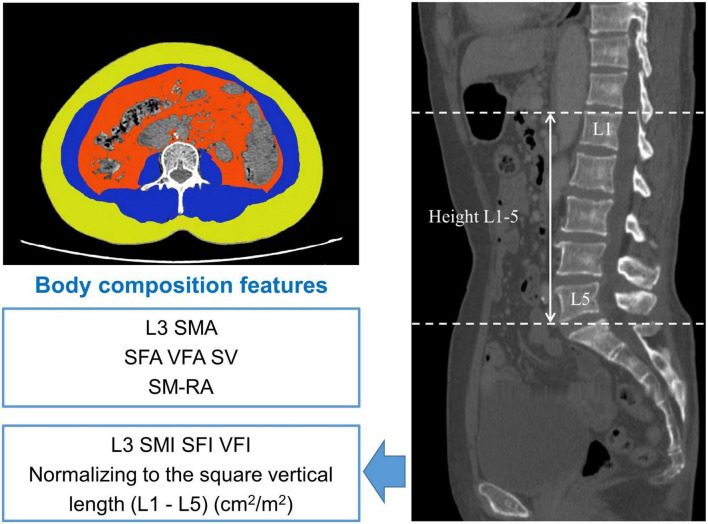
Body composition measurement of the third lumbar vertebra region. Yellow region: subcutaneous fat area (SFA) (cm^2^); blue region: L3 lumbar skeletal muscle area (L3 SMA) (cm^2^), red region: visceral fat area (VFA) (cm^2^). SFI, subcutaneous fat index; SM-RA, skeletal muscle radiation attenuation; SV, subcutaneous and visceral ratio; L3 SMI, L3 skeletal muscle index; VFI, visceral fat index.

We carefully analyzed CT images at the third lumbar vertebra (L3) using *X-SECTION functions in 3D reformat software (GE AW4.6 workstations, GE Healthcare).* Then, we further determined the muscle area, including L3 skeletal muscle area, VFA (cm^2^), SFA (cm^2^), and SM-RA. Examples of the areas of SFA, skeletal muscle area (SMA), and VFA are described in Figure 1. The L3 SMI, VFI, and SFI were further calculated by normalizing the related index to height 1–5 and reported as cm^2^/m^2^ (36, 37). To make the data presentation convenient and neat, the true values of SMI, SFI, and VFI are expressed by numerical value * 10^2^. Moreover, we defined SV as the subcutaneous and visceral ratio (38).

### Endpoints

The primary endpoint of our study was overall survival (OS), which is defined as the time from the date of the radical gastrectomy until death. The last follow-up visit was in December 2021. Moreover, we defined the cutoff points for L3 SMI, which would best predict the mortality in our present study. This optimum stratification method has been previously used to determine the threshold value which separated two categories (sarcopenia and non-sarcopenia) (24). We used this method to find the most significant *p*-value by using the log-rank test to define the sex-specific cutoff points associated with OS in patients. In addition, VFA, VFI, SFA, SFI, SV, and SM-RA were also classified into two groups and examined using the optimum stratification method same as L3 SMI.

### Statistical analyses

The results from the clinical trial were conducted by the statistical computer software of IBM SPSS Statistics 24.0 and R 4.1.3. For analysis of baseline clinical characteristics between sarcopenia and non-sarcopenia, the independent sample *t*-test was used for continuous variables and Pearson’s chi-square test for categorical data. The measurement data were expressed as mean ± SD (standard deviation), while the count data were expressed as numbers with percentages. The receiver operating characteristic curve (ROC) analysis was performed to define the cutoff point of L3 SMI, SFA, SFI, VFA, VFI, SV, and SM-RA. Kaplan–Meier method was used to estimate the OS, and the log-rank test was used to evaluate the differences between curves. Variables with *p* < 0.2 were included in the multivariate analysis as a previous study (39). Multivariate analysis was performed using Cox’s proportional hazard model to assess the importance of potential prognostic factors. *P*-value < 0.05 was considered significant.

## Results

### Flowchart

**FIGURE 2:**
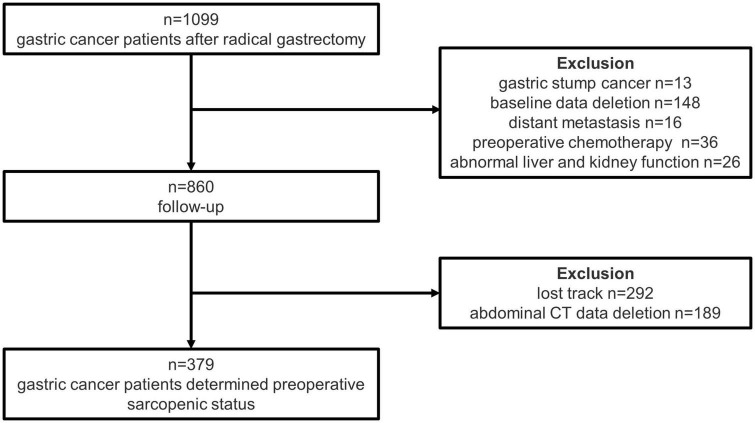
Flow diagram of patients.

Of the 1,099 GC patients after radical gastrectomy at the Department of gastrointestinal surgery, Xiangya Hospital of Central South University between January 2011 and December 2016 was collected. After excluding 13 patients with gastric stump cancer, 148 patients with baseline data deletion, 16 patients with distant metastasis, 36 patients with preoperative chemotherapy, and 26 patients with abnormal liver and kidney function, 860 patients enrolled in follow-up. We further ruled out patients who lost track (*n* = 292) and without abdominal CT data (*n* = 189), after which a total of 379 patients with GC were included in our study to determine their preoperative sarcopenic status (as shown in Figure 2).

### Define of sarcopenia

**FIGURE 3:**
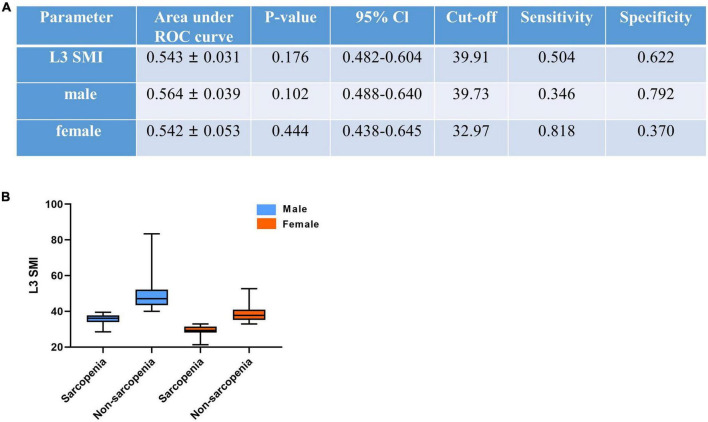
CT-determined sarcopenic status **(A)** according to sex-specific L3 skeletal muscle index (L3 SMI) cutoffs for sarcopenia **(B)**. L3 SMI, L3 skeletal muscle index. The cutoff of SMI was reduced by 100 times for convenience.

After ROC analysis, we defined sex-specific and SMI-related mortality cutoff as 39.73 and 32.97 cm^2^/m^2^ for men and women, respectively (shown in Figure 3). Sarcopenia patients were classified according to these cutoff values for men and women. The average L3 SMI in all cases was 41.91 cm^2^/m^2^ (Table 1). After classification, our results showed that the sex-specific average was 45.04 cm^2^/m^2^ for men and 35.54 cm^2^/m^2^ for women.

**TABLE 1:** Baseline clinical characteristics and comparisons between patients with CT-determined sarcopenia and non-sarcopenia.

Parameter	Total (*n* = 379)	Non-sarcopenia (*n* = 277)	Sarcopenia (*n* = 102)	*P-value*
Age (years)	56.65 ± 10.68	56.36 ± 9.94	57.45 ± 12.51	0.428
Gender				0.283
Male	254	190 (74.80%)	64 (25.20%)	
Female	125	87 (69.60%)	38 (30.40%)	
Type of gastrectomy				0.211
Total	55	44 (80.00%)	11 (20.00%)	
Subtotal	324	233 (71.91%)	91 (28.09%)	
Degree of differentiation				0.208
Poor	278	210 (75.54%)	68 (24.46%)	
Medium and high	69	47 (68.11%)	22 (31.88%)	
Pathological TNM				0.068
I	77	53 (68.83%)	24 (31.17%)	
II	120	97 (80.83%)	23 (19.17%)	
III	182	127 (69.78%)	55 (30.22%)	
Depth of invasion				0.752
T1	67	46 (68.66%)	21 (31.34%)	
T2	65	50 (76.92%)	15 (23.08%)	
T3	115	85 (73.91%)	30 (26.09%)	
T4	132	96 (72.73%)	36 (27.27%)	
Lymph node metastasis				**0.036**
N0	128	94 (73.44%)	34 (26.56%)	
N1	74	57 (77.03%)	17 (22.97%)	
N3	73	60 (82.19%)	13 (17.81%)	
N4	104	66 (63.46%)	38 (36.54%)	
RBC (10^12^/L)	4.03 ± 0.66	4.08 ± 0.67	3.85 ± 0.57	0.052
WBC (10^9^/L)	6.07 ± 1.93	6.14 ± 2.00	5.87 ± 1.74	0.228
PLT (10^9^/L)	229.02 ± 73.32	232.54 ± 73.20	219.62 ± 73.19	0.129
AST (U/L)	23.23 ± 12.48	22.95 ± 12.50	23.99 ± 12.44	0.476
ALT (U/L)	20.49 ± 16.55	20.86 ± 16.54	19.49 ± 16.63	0.479
Cr (μmol/L)	85.04 ± 16.65	85.90 ± 16.54	82.70 ± 16.80	0.099
Height 1–5 (cm)	16.83 ± 1.02	16.64 ± 1.02	17.33 ± 0.86	**0.000**
SMA (cm^2^)	118.71 ± 25.78	125.39 ± 24.97	100.57 ± 18.12	**0.000**
L3 SMI (cm^2^/m^2^) ×10^2^	41.91 ± 8.38	45.08 ± 7.23	33.29 ± 4.14	**0.000**
VFA (cm^2^)	61.51 ± 42.60	66.02 ± 44.27	49.27 ± 35.03	**0.000**
VFI (cm^2^/m^2^) ×10^2^	21.93 ± 15.21	23.92 ± 15.85	16.52 ± 11.82	**0.000**
SFA (cm^2^)	95.32 ± 60.82	103.22 ± 60.83	73.87 ± 55.65	**0.000**
SFI (cm^2^/m^2^) ×10^2^	34.33 ± 23.07	37.67 ± 23.21	25.24 ± 20.15	**0.000**
SV	1.75 ± 1.09	1.80 ± 1.09	1.62 ± 1.09	0.136
SM-RA (HU)	44.03 ± 22.89	44.83 ± 26.22	41.87 ± 8.67	0.265

Data were shown as MEAN ± SD. AST, aspartate aminotransferase; ALT, alanine aminotransferase; Cr, creatinine; HU, Hounsfield units; PLT, platelet; RBC, red blood cell; SFA, subcutaneous fat area; SFI, subcutaneous fat index; SMA, skeletal muscle area; SM-RA, skeletal muscle radiation attenuation; SV, subcutaneous and visceral ratio; L3 SMI, L3 skeletal muscle index; VFA, visceral fat area; VFI, visceral fat index; WBC, white blood cell. Significant P-values are indicated in bold.

### Baseline characteristics

As shown in Table 1, the average age in all patients was 56.65 ± 10.68 years, with predominantly male patients (254, 67.02%). As for lymph node metastasis, there is a significant difference in its proportion between the two groups (*p* < 0.05). No significant difference was found in age, gender, type of gastrectomy, degree of differentiation, pathological stages, depth of invasion, and laboratory data between sarcopenia and non-sarcopenia. The prevalence of sarcopenia was 25.20% in men and 30.40% in women. However, increased height 1–5 (*p* < 0.001) was found in patients with sarcopenia. Notably, the assessment indexes, such as SMA (*p* < 0.001), L3 SMI (*p* < 0.001), VFA (*p* < 0.001), VFI (*p* < 0.001), SFA (*p* < 0.001), and SFI (*p* < 0.001), were significantly decreased in patients with sarcopenia.

### Receiver operating characteristic curve analysis

Using the same method of SMI, we defined the cutoff values for VFA (87.10 cm^2^), VFI (15.52 cm^2^/m^2^), SFA (141.55 cm^2^), SFI (26.46 cm^2^/m^2^), SMA (106.72 cm^2^), SV (1.478), and SM-RA (46.15 HU), respectively (Table 2). The features of patients with sarcopenia and non-sarcopenia are also shown in Table 1.

**TABLE 2:** ROC analysis.

Parameter	Area under ROC curve	*P-value*	95% Cl	Cut-off	Sensitivity	Specificity
VFA	0.511 ± 0.032	0.734	0.448–0.573	87.10	0.278	0.776
Male	0.529 ± 0.039	0.453	0.453–0.606	49.68	0.593	0.526
Female	0.547 ± 0.056	0.388	0.436–0.657	48.37	0.500	0.654
VFI	0.507 ± 0.032	0.831	0.445–0.569	15.52	0.643	0.417
Male	0.521 ± 0.039	0.586	0.445–0.598	15.52	0.630	0.486
Female	0.539 ± 0.056	0.472	0.428–0.650	17.13	0.455	0.667
SFA	0.524 ± 0.031	0.448	0.463–0.585	141.55	0.816	0.257
Male	0.520 ± 0.039	0.602	0.444–0.596	82.81	0.506	0.595
Female	0.636 ± 0.054	**0.012**	0.530–0.742	79.68	0.386	0.889
SFI	0.521 ± 0.031	0.506	0.460–0.582	26.46	0.448	0.613
Male	0.512 ± 0.039	0.752	0.436–0.589	30.67	0.469	0.636
Female	0.615 ± 0.054	**0.034**	0.509–0.722	31.06	0.409	0.864
SMA	0.525 ± 0.032	0.436	0.463–0.589	106.72	0.400	0.673
Male	0.525 ± 0.040	0.524	0.447–0.602	115.67	0.309	0.792
Female	0.524 ± 0.054	0.653	0.418–0.631	90.77	0.614	0.481
SV	0.522 ± 0.031	0.486	0.461–0.583	1.478	0.560	0.530
Male	0.504 ± 0.039	0.923	0.428–0.579	1.056	0.704	0.370
Female	0.608 ± 0.055	**0.046**	0.500–0.716	2.242	0.659	0.580
SM-RA	0.601 ± 0.030	**0.001**	0.542–0.659	46.15	0.770	0.429
Male	0.624 ± 0.037	**0.001**	0.552–0.697	44.15	0.617	0.613
Female	0.540 ± 0.052	0.463	0.437–0.642	46.20	0.886	0.272

SFA, subcutaneous fat area; SFI, subcutaneous fat index; SMA, skeletal muscle area; SM-RA, skeletal muscle radiation attenuation; SV, subcutaneous and visceral ratio; VFA, visceral fat area; VFI, visceral fat index. The cutoff of SFI and VFI was reduced by 100 times for convenience. Significant P-values are indicated in bold.

### Prognostic factors

The results of univariate analysis are shown in Table 3. On the basis of univariate analysis, we found that the pathological TNM (II and III) [hazard ratio (HR), 4.424; 95% CI, 1.723–11.355; *p* = 0.002; HR, 12.231; 95% CI, 4.981–30.035; *p* = 0.000], depth of invasion [T3-4] (HR, 4.810; 95% CI, 2.894–7.994; *p* = 0.000), lymph node metastasis (N2–3) (HR, 3.091; 95% CI, 2.165–4.313; *p* = 0.000), poor differentiation (HR, 1.635; 95% CI, 1.079–2.479; *p* = 0.045), sarcopenia (men) (HR, 1.803; 95% CI, 1.092–2.977; *p* = 0.007), SMA (men) (HR, 1.601; 95% CI, 1.018–2.516; *p* = 0.041), L3 SMI (HR, 1.538; 95% CI, 1.094–2.162; *p* = 0.010), SFA (especially for women) (HR, 2.931; 95% CI, 1.641–5.235; *p* = 0.000), SFI (especially for women) (HR, 2.756; 95% CI, 1.344–5.655; *p* = 0.000), SV (especially for women) (HR, 1.983; 95% CI, 1.136–3.461; *p* = 0.016), SM-RA (HR, 2.100; 95% CI, 1.497–2.947; *p* = 0.000), and SM-RA (especially for men) (HR, 2.232; 95% CI, 1.465–3.401; *p* = 0.001) were statistically significant. After multivariate analysis, pathological TNM (III), depth of invasion (T3–4), poor differentiation, sarcopenia (especially for men), SFA (especially for women), and SM-RA (both for men and women) were shown to be significant independent predictors of OS.

**TABLE 3:** Results of Cox regression analysis for overall survival.

Parameter	Univariable		Multivariable	
	HR (95% CI)	*P*	HR (95% CI)	*P*
Age (> 65 years)	1.255 (0.819–1.924)	0.262		
Gender (female)	1.180 (0.827–1.684)	0.347		
**Pathological TNM**				
II	4.424 (1.723–11.355)	**0.002**	2.347 (0.817–6.742)	0.113
III	12.231 (4.981–30.035)	**0.000**	5.385 (1.833–15.823)	**0.002**
Depth of invasion (T3–4)	4.810 (2.894–7.994)	**0.000**	1.869 (1.003–3.484)	**0.049**
Lymph node metastasis (N2–3)	3.091 (2.165–4.313)	**0.000**		
Poor differentiation	1.635 (1.079–2.479)	**0.045**	1.681 (1.032–2.739)	**0.037**
CT-determined sarcopenia	1.185 (0.809–1.736)	0.363		
CT-determined sarcopenia (male)	1.803 (1.092–2.977)	**0.007**	1.719 (1.107–2.671)	**0.016**
CT-determined sarcopenia (female)	0.515 (0.285–0.930)	0.055		
SMA (<106.72)	1.395 (0.995–1.956)	0.054		
SMA (male < 115.67)	1.601 (1.018–2.516)	**0.041**		
SMA (female < 90.77)	0.713 (0.403–1.262)	0.246		
L3 SMI (<39.91 × 10^2^)	1.538 (1.094–2.162)	**0.010**		
VFA (<87.10)	0.816 (0.551–1.208)	0.283		
VFA (male < 49.68)	0.667 (0.440–1.010)	0.058		
VFA (female < 48.37)	1.388 (0.784–2.457)	0.243		
VFI (<15.52 × 10^2^)	0.767 (0.547–1.076)	0.135		
VFI (male < 15.52 × 10^2^)	0.650 (0.422–1.003)	0.051		
VFI (female < 17.13 × 10^2^)	1.254 (0.706–2.230)	0.426		
SFA (<141.55)	1.265 (0.858–1.865)	0.263		
SFA (male < 82.81)	0.734 (0.483–1.117)	0.142		
SFA (female < 79.68)	2.931 (1.641–5.235)	**0.000**	3.441 (1.902–6.244)	**0.000**
SFI (< 26.46 × 10^2^)	1.167 (0.831–1.640)	0.364		
SFI (male < 30.67 × 10^2^)	0.705 (0.460–1.081)	0.097		
SFI (female < 31.06×10^2^)	2.756 (1.344–5.655)	**0.000**		
SV (<1.478)	1.287 (0.922–1.795)	0.136		
SV (male < 1.056)	0.813 (0.526–1.257)	0.366		
SV (female < 1.056)	1.983 (1.136–3.461)	**0.016**		
SM-RA (<46.15)	2.100 (1.497–2.947)	**0.000**	2.174 (1.442–3.278)	**0.000**
SM-RA (male < 44.15)	2.232 (1.465–3.401)	**0.001**	2.187 (1.423–3.361)	**0.000**
SM-RA (female < 46.20)	1.791 (0.948–3.384)	0.123	2.337 (1.079–5.062)	**0.031**

CI, confidence interval; HR, hazard ratio; SFA, subcutaneous fat area; SFI, subcutaneous fat index; SMA, skeletal muscle area; SM-RA, skeletal muscle radiation attenuation; SV, subcutaneous and visceral ratio; L3 SMI, L3 skeletal muscle index; VFA, visceral fat area; VFI, visceral fat index. Significant P-values are indicated in bold.

### Long-term results

**FIGURE 4:**
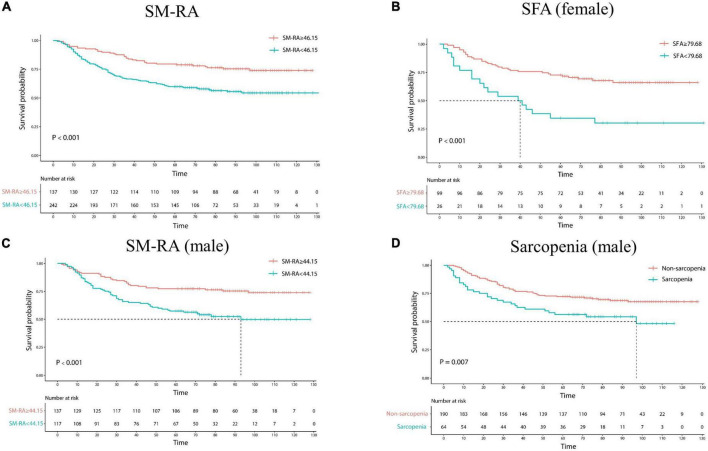
Kaplan–Meier estimates of overall survival in patients with gastric cancer according to SM-RA **(A)**, SFA status for female **(B)**, SM-RA for male **(C)**, and CT-determined sarcopenia for male **(D)**. SFA, subcutaneous fat area; SM-RA, skeletal muscle radiation attenuation.

As shown in Table 3 and Figure 4, Advanced GC patients had worse survival, which is reflected in advanced pathological TNM stage, depth of invasion, and lymph node metastasis. Moreover, GC patients with poor differentiation have a lower 5-year survival rate (64.75% vs. 73.91%). L3 SMI and SM-RA were also significantly associated with OS in GC patients, and the 5-year OS rates were better in the high level of L3 SMI (60.38% vs. 71.82%) and SM-RA (79.56% vs. 59.92%) group.

In addition, the 5-year OS rate was significantly lower in the sarcopenia group (56.25%) than in the non-sarcopenic (72.11%) men. High level of SM-RA had better 5-year OS rate for male patients (77.37% vs. 57.27%). Meanwhile, for female GC patients, high levels of SFA, SFI, and SV showed better 5-year OS rates (72.73% vs. 34.62%; 72.92% vs. 37.93%; and 75.81% vs. 53.97%).

## Discussion

In the present study, the prevalence of preoperative sarcopenia in patients with GC was 25.20% in men, which is dramatically lower than in women (30.40%). In terms of long-term outcomes, 5-year OS was significantly shorter in patients with sarcopenia. Moreover, in addition to indicators that evaluate the disease progression of cancer (pathological TNM, depth of invasion, lymph node metastasis, poor differentiation), preoperative sarcopenia (for men), decreased SMA (for men), SFA, SFI, SV (for women), and SM-RA (especially for men) were also significantly associated with lower 5-year OS in GC patients. Multivariate analysis revealed that pathological TNM, depth of invasion, poor differentiation, and SM-RA were independent prognostic factors for patients with GC who underwent radical gastrectomy. Notably, the present study demonstrated the predictive effect of sarcopenia on survival outcomes is depended on sex.

Although previous studies came to heterogeneous conclusions, sarcopenia was generally identified as an independent prognostic factor for a poor OS of GC. In a retrospective study, CT-determined sarcopenia was reported to be an independent prognostic factor for patients with advanced GC who underwent palliative chemotherapy in Korea (16). In Japan, scholars also came to a similar conclusion that preoperative sarcopenia was related to poor survival in GC patients and appeared to be a significant negative prognostic factor in patients with GC who underwent curative resection (40). A cohort study from China also drew the similar conclusion that sarcopenia was an independent predictor of severe postoperative complications and long-term survival after radical gastrectomy (41). This conclusion is applicable to Western patients (42). Moreover, preoperative sarcopenia is also independently associated with severe postoperative complications in GC patients undergoing gastrectomy (43–46), and an independent prognostic factor for progression-free survival (PFS) in patients with microsatellite-stable (MSS) GC treated with programmed death-1 (PD-1) inhibitors (47). However, for patients with GC who received neoadjuvant chemotherapy, sarcopenia did not appear to impact the length of stay or survival (48). Similarly, in a study from the Netherlands, sarcopenia was not associated with postoperative morbidity or mortality in GC surgical patients (49).

The diagnostic criteria for sarcopenia remain controversial and the cutoff values used for defining sarcopenia were different between studies. In the studies described above, the Korean study defined sarcopenia as L3 SMI ≤ 49 cm^2^/m^2^ for men and ≤ 31 cm^2^/m^2^ for women using Korean-specific cutoff values (16). However, the diagnostic criteria for sarcopenia in the Japan study were defined as a decreased arm muscle area < 38.05 cm^2^ in men and < 27.87 cm^2^ in women combined with a decline in grip strength < 26 kgf in men and < 18 kgf in women (40). The cohort from China calculated the cutoff values of sarcopenia and classified L3 SMI as < 34.9 cm^2^/m^2^ for women and < 40.8 cm^2^/m^2^ for men (41). In our present study, we defined sex-specific L3 SMI-related mortality cutoff as 39.73 and 32.97 cm^2^/m^2^ for men and women, respectively, which is consistent with previous studies, that cutoff values for L3 SMI to define sarcopenia in GC ranged from 32.5 to 54.5 cm^2^/m^2^ in men and from 28.6 to 41.0 cm^2^/m^2^ in women (14). More research is needed to obtain good reference values for various ethnic groups and cancer patients.

As we described before, both classic indicator L3 SMI and other indicators like fat area, including both fat area in visceral or subcutaneous, VFI, SFI, SV, and SM-RA, were taken into account in the present study. A recent study attempted to investigate more accurately predictive factors of postoperative complications or prognosis in patients with gastric cancer rather than sarcopenia defined by L3 SMI. The authors found that both psoas muscle mass index (PMI) and VFA were useful predictive factors for postoperative pneumonia and intra-abdominal abscess in patients with gastric cancer, but only PMI was a useful prognostic factor (34). Rinninella et al. assessed body composition changes of GC patients receiving the docetaxel (FLOT) regimen and reported that preoperative FLOT was associated with a further reduction in both SMI and VFI. However, these changes were not associated with short-term outcomes (32). Our study came to a similar conclusion that neither VFA nor VFI was associated with long-term results in patients with GC. Although visceral fat does not seem to affect the prognosis of GC patients, subcutaneous fat plays a role in GC prognosis according to our results. Furthermore, SFA, SFI, and SV in women are prognostic factors for the 5-year OS of GC patients who underwent radical gastrectomy. Similarly, a retrospective from Japan demonstrated that visceral-to-subcutaneous adipose tissue area ratio (VSR) was a prognostic factor for the overall survival of elderly patients with gastric cancer after gastrectomy (33). Moreover, reduced SM-RA is independently associated with postoperative outcomes in cancer patients (35, 50), which is consistent with our findings.

The influence of sex factor on the relationship between sarcopenia and the prognosis of cancer is an emerging field. Women exhibit higher global fat mass and reduced skeletal muscle mass (51). One reason for the sex difference might be the discrepancy in muscle and fat mass between men and women, since testosterone level which has an anabolic effect on the muscle is higher in men (29). Skeletal muscle fiber composition, myosin heavy chain expression, contractile function, and the regulation of these physiological differences by thyroid hormone, estrogen, and testosterone might also play important role in sex differences (30). Moreover, the absolute rate of decline in skeletal muscle mass was reported to be greater in men than women (27). Similarly, accumulating studies demonstrated that male cancer patients lost more body weight, muscle mass, and had higher rates of mortality compared with female patients (52, 53). A recent study demonstrated that surgery-induced sarcopenia was a risk factor for OS and relapse-free survival (RFS) in men only (26). Consistent with previous studies, our result also showed that sarcopenia was a risk factor for 5-year OS only in men. However, SFA, SFI, and SV in women were prognostic factors for the 5-year OS, and SFA was significantly associated with 5-year OS in GC patients after multivariable analysis in women rather than men. As previously mentioned, women are characterized by an increased propensity to store subcutaneous fat (51), which might partly explain why subcutaneous fat is more sensitive in women than men. Moreover, low SMI in men and high visceral-to-SFA ratio in women were good prognosticators for patients with colorectal cancer (54). We speculated that muscle reserves in men are more important than fat reserves for the pathological of cancer, which is different from women. However, the potential mechanism of this sex difference needs to be further studied.

One limitation of this study was that we were unable to collect information about muscle strength and physical performance since our study is a retrospective study. Similarly, no record of the height information of patients was found in the computerized database; thus, we have to use the height 1–5 obtained from CT scans instead. Lastly, the sample size is not large enough due to the high rate of lost follow-up. A prospective large cohort study is needed to verify the results.

## Conclusion

Decreased L3 SMI and SM-RA were associated with increased mortality in patients with GC after radical gastrectomy. The impact of indexes investigated in our study on survival outcomes is dependent on sex. The association between sarcopenia, SMA, SM-RA, and long-term survival was meaningful in men but not in women. On the contrary, SFA, SFI, and SV were significantly associated with 5-year OS in GC patients in women rather than men. Further studies are needed to clarify the specific mechanisms of sex difference.

## Data availability statement

The raw data supporting the conclusions of this article will be made available by the authors, without undue reservation.

## Ethics statement

The studies involving human participants were reviewed and approved by the Ethics Committee of Xiangya Hospital of Central South University. The patients/participants provided their written informed consent to participate in this study.

## Author contributions

TL, JG, and XY performed the material preparation, data collection, and analysis. MT, HL, and TL wrote the first draft of the manuscript. MT, TL, XY, and JG reviewed and edited the previous versions of the manuscript. JZ, FT, and KS verified the data and amended the manuscript. All authors contributed to the study conception and design, read, and approved the final manuscript.
